# Fluorescence spectroscopy and chemometrics for simultaneous monitoring of cell concentration, chlorophyll and fatty acids in *Nannochloropsis oceanica*

**DOI:** 10.1038/s41598-020-64628-7

**Published:** 2020-05-06

**Authors:** Marta Sá, Carlo G. Bertinetto, Narcís Ferrer-Ledo, Jeroen J. Jansen, Rene Wijffels, João G. Crespo, Maria Barbosa, Claudia F. Galinha

**Affiliations:** 1 LAQV-REQUIMTE, Chemistry Department, FCT, Universidade Nova de Lisboa, Caparica, Portugal; 2 Bioprocess Engineering, AlgaePARC, Wageningen University and Research, Wageningen, Netherlands; 3 Radboud University, Department of Analytical Chemistry and Chemometrics, Institute for Molecules and Materials, Nijmegen, Netherlands

**Keywords:** Fluorescence spectroscopy, Biosynthesis, Mathematics and computing, Data processing, Sensors, Biological techniques

## Abstract

Online monitoring of algal biotechnological processes still requires development to support economic sustainability. In this work, fluorescence spectroscopy coupled with chemometric modelling is studied to monitor simultaneously several compounds of interest, such as chlorophyll and fatty acids, but also the biomass as a whole (cell concentration). Fluorescence excitation-emission matrices (EEM) were acquired in experiments where different environmental growing parameters were tested, namely light regime, temperature and nitrogen (replete or deplete medium). The prediction models developed have a high R^2^ for the validation data set for all five parameters monitored, specifically cell concentration (0.66), chlorophyll (0.78), and fatty acid as total (0.78), saturated (0.81) and unsaturated (0.74). Regression coefficient maps of the models show the importance of the pigment region for all outputs studied, and the protein-like fluorescence region for the cell concentration. These results demonstrate for the first time the potential of fluorescence spectroscopy for *in vivo* and real-time monitoring of these key performance parameters during *Nannochloropsis oceanica* cultivation.

## Introduction

Microalgae industrial production is still a niche industry, although efforts are being done to improve the economic viability of the overall process^1,2^. Online monitoring through spectroscopic techniques is already a reality in several other bio-based industries^3–6^. Development of an appropriate tool able to monitor several metabolites simultaneously would be a great advantage. Nowadays, biological parameters are monitored off-line, where a sample is withdrawn, from the cultivation or from the process in the biorefinery, to be analysed. These analyses can take some minutes or a couple of hours, or days, depending on the parameter to be measured and techniques involved. For example, cell concentration is a rather fast and simple method, while assessing metabolites’ content is much slower, because extraction steps and chromatographic techniques are often needed. Fluorescence spectroscopy represents a viable solution since it is a non-invasive and non-destructive technique that already proved its value in other industries, like the food industry^3–5^, or even in wastewater treatment plants that are characterised for being complex biological systems^6–8^. This technique is able to detect natural fluorophores but is also sensitive to interactions between fluorophores and non-fluorescent compounds, increasing the range of compounds that can be monitored by it^9^. Additionally, with the improvement of chemometric tools, it is possible to extract more information from such signals.

*Nannochloropsis oceanica* is a promising microalga due to its ability to produce high amount of lipids. This microalga is cultivated in sea water, which has been pointed as the most sustainable solution for microalgae production^2,10,11^. The most common product of a microalgae industry is still the whole biomass, being of extreme importance to monitor cell concentration^12^. It is also known that chlorophyll is an important pigment in several industries^13,14^, and being a oleaginous microalga, there are an increasing interest in industrializing *N. oceanica* to produce high quantities of lipids, but also high nutritional value oils^2,10^.

Previous studies demonstrated the advantage of using fluorescence spectroscopy in a microalgae biorefinery context using *Dunaliella salina*^15–17^. This microalga is also a saline microalga capable of producing high contents of carotenoids, being already produced at industrial scale for it. Fluorescence spectroscopy coupled with chemometric tools was used to develop prediction models for several parameters, such as cell concentration and viability, carotenoids content and nitrate concentration for *D. salina* upstream processes^15–17^. Cell concentration of *Chlorella vulgaris* and *Spirulina* sp. was also determined by Shin *et al*. using a *in situ* fluorometry technique^18^.

More recently, a new study from the authors, showed that eicosapentaenoic acid (EPA) can be also monitored using fluorescence spectroscopy in N. oceanica cultures^19^. Therefore, the goal of this work was to develop models to predict simultaneously cell concentration, chlorophyll and fatty acid content in *N. oceanica*, based only on fluorescence spectra acquired directly from *in vivo* culture broths. Furthermore, the spectral regions with higher relevance for the prediction models of all parameters were also studied, providing a better understanding of how the fluorescence can be useful to monitor fluorophores and non-fluorophores molecules. The development of this monitoring tool, able to be used online and based on a non-destructive technique will enhance the knowledge about the culture at real time, during the cultivation process, contributing for an increase competitiveness of microalgae industry.

## Material and Methods

### Nannochloropsis oceanica pre-culture and experiments

*Nannochloropsis oceanica* NCT02 was provided by NECTON, S.A. (Olhão, Portugal). The *N. oceanica* inoculum was kept in 250 mL Erlenmeyer flasks, under the follow conditions: 25 °C, 90 rpm in an orbital shaker, 100 µmol/m^2^.s of incident light, day/night cycle (16/8 hours) light regime, and 0.2% CO_2_. The cultivation media contained natural sea water (from Eastern Scheldt, the Netherlands) filtered (0.2 µm) and supplemented with 10.7 mM of NaNO_3_, 0.535 mM of KH_2_PO_4_. NUTRIBLOOM from PhytoBloom, a nutrient solution, and HEPES buffer (20 mM) were added to the media and the pH set to 7.8. Medium sterilization was performed by cellulose acetate membrane filtration (using SARTOBRAN Capsule with 0.2 µm of pore size, Sartorius) directly into the Erlenmeyer or bioreactor. Experiments were performed in batch mode, in a heat-sterilized flat-panel, with a 1.8 L of working volume and a light path of 20.7 mm (Labfors 5 Lux, Infors HT, Switzerland, 2010). The light was provided by LED lamps (28 V, 600 Watt) with warm spectrum (450–620 nm). In the beginning of the experiment light was set at 200 µmol/m^2^.s, and increased to 636 µmol/m^2^.s when the back light reached 50 µmol/m^2^. The culture homogenization was done by filter sterilized air in an airlift-loop at a flow rate of 1 L/min, and the pH was controlled by CO_2_ injection. The bioreactor temperature was controlled by water-jacket.

Three cultivation parameters were tested in eight experiments (Table 1): light regime, temperature, and nitrogen supply – with (√) or without (X) nitrogen. Light regime was set in the beginning of the experiment and two approaches were tested: 24 hours of light or 16 h of light and 8 h of dark (d/n cycle). Temperature was set in the beginning (15, 20, 25 and 30 °C) and kept through the experiment; in one batch, the temperature was decreased from 25 to 15 °C when a light supply rate of 1 × 10^–13^ µmol/cell.s was reached. All batches started with a replete nitrogen medium to enable biomass growth. For six of the eight batches (Table 1, Nitrogen supply “X”), a second step, the nitrogen depletion phase, was performed. Briefly, the biomass was collected, centrifuged (2500 rpm, 15 minutes) and washed with nitrogen deplete medium, and the bioreactor was then refilled with culture and nitrogen deplete medium until reaching a specific light supply rate of 1 × 10^−13^ µmol/cell.s. More detailed information about the experiments are available in Sá *et al*.^19^.

**Table 1:** Experimental conditions of the eight batch experiments performed.

Temp (°C)	Nitrogen supply^(a)^	Light cycle (hours)
15	X	d (24)
20	X	d (24)
25^(b)^	X	d (24)
25	X	d/n (16/8)
25	√	d/n (16/8)
25 → 15	√	d/n (16/8)
30	X	d (24)

Three different environmental growing parameters were tested, namely temperature, nitrogen supply. (^(a)^X = absent; √ = present;) and light cycle (d (24): 24 h of light; or d/n (16/8): 16 h of light and 8 h of dark). ^(b)^This batch was performed twice.

### Offline measurements

Samples were taken every day to measure cell concentration, chlorophyll content, fatty acid composition and spectrofluorescence.

Cell concentration was measured in a MULTISIZER II (Beckman Counter), in duplicates, using a 50 µm aperture tube and Isotone II diluent to dilute the samples.

Chlorophyll content was assessed by a spectrophotometric method as described by Leu and Hsu^20^. An aliquot of 2 mL was centrifuged (5000 g, 5 min) and stored at −80 °C until further analysis. Extraction was performed with 2 mL of methanol, samples were sonicated for 5 min and incubated for 40 min at 60 °C, following by cooling for 15 min in ice. Extraction steps were repeated until a white pellet was recovered. The modified Arnaud equation was used to calculate chlorophyll content:

$$Chla=(16.72\times {A}_{665}-9.16\times {A}_{652})\times dilution\,factor(mg/L)$$

Lipid composition was measured in lyophilized biomass samples, previously washed with 0.5 M ammonium formate, as described by Breuer *et al*. and Leon-Saiki^21,22^. Briefly, 10 mg of sample were disrupted by beat beater and an extraction was performed with chloroform:methanol (1:1.25, v-v), containing the internal standards for triacylglycerol (TAG) and polar (PL) fractions, 170 µg/mL of tripentadecanoin (9:0) and 170 µg/mL of 1,2-dipentadecanoyl-sn-glycero-3-[phosphor-rac-(1-glycerol)] (sodium salt) (15:0) respectively. TAG and PL were then separated in a SPE silica gel column (Sep-Pak Vac 6cc, Waters) using hexane:diethylether (7:1, v-v) and methanol:acetone:hexane (2:2:1, v-v) respectively. Methylation was performed in both fractions prior to quantification by gas chromatography (GC-FID). The results were calculated as percentage of total, saturated and unsaturated fatty acids in a dry weight basis.

Fluorescence spectra were acquired in a Shimadzu RF-6000 spectrofluorophotometer. The samples were placed in a cuvette and no sedimentation was observed during the spectra acquisition, which occurred in 5 minutes. The excitation-emission matrices (EEMs) obtained ranged from 250 to 790 nm for excitation wavelengths, and between 260 and 800 nm for emission wavelengths, in steps of 5 nm. Excitation and emission monochromator slit widths were set at 3 nm, with a scan speed of 12000 nm/min.

### Chemometric models development

The EEMs obtained during the eight experiments were combined and pre-processed together using drEEM toolbox (http://www.models.life.ku.dk/dreem)^23^. Rayleigh scatter of first order was removed and replaced by empty values; the second order was replaced with an interpolation of surrounding data points^23^. Any fluorescence signal corresponding to emission wavelengths (y-axis) shorter than the excitation wavelengths (x-axis) was replaced by zeros. Inner filter effects, i.e. whenever excessive concentration causes re-absorption within the sample and thus a non-linear fluorescence response, were accounted for using a correction matrix derived from the sample’s absorbance spectrum^24^.

The pre-processed EEMs were correlated with five biological parameters (cell concentration, chlorophyll and fatty acids content as total, saturated and unsaturated) using Projection to Latent Structures (PLS) regression. This method finds linear combinations of the observed variables (i.e. the excitation-emission wavelengths, the inputs) that yield the best linear regression to the predicted variables (i.e. the biological parameters, the outputs). These linear combinations are known as Latent Variables (LVs), and can be seen as underlying structures or patterns that are correlated to the predicted parameters more directly than the original spectral variables measured by the experiment. More extensive descriptions of PLS can be found elsewhere^25^. In the current work, the multiway version of PLS, known as N-PLS^26^, was used, which allows for fully exploiting the mathematical relationships among the three modes of the EEM data (i.e. sample, excitation, emission).

To facilitate the modelling task, the predicted variables were converted into their logarithm with base 10 to normalise their distribution. The predictive models were validated by a 4-fold double cross-validation^27^. In short, the data set was split randomly into a training and validation set, consisting of 75% and 25% of the total data, respectively. The training set was used to calibrate the model and optimise the number of LVs, using a leave-one-out cross-validation (LOOCV), in which one sample from the training set was removed, a PLS model was built on the remaining training samples and assessed for the left-out sample; this procedure was then repeated with a different leave-out sample until all samples have been rotated. The external validation set was held out of this loop and was used to validate the model with the optimal number of LVs as determined by the LOOCV. The whole procedure (LOOCV + external validation) was repeated three more times, using the external validation data that was previously used for training, until every sample has appeared in the external validation set once.

Several parameters were evaluated to assess model’s quality: the variance explained in the biological parameters (%), the root mean square error of cross-validation (RMSECV) and prediction (RMSEP), and the R^2^ and slopes of the training and external validation (from now on mentioned as validation) sets.

After evaluating the quality of the models, a final model was built for each predicted parameter using the whole data set, using the optimal number of LVs defined by the previous validation, in order to determine and examine the regression coefficient values.

All multivariate statistical analysis were performed in MATLAB (The MathWorks) using the drEEM toolbox (http://www.models.life.ku.dk/dreem) and n-way toolbox^26,28^.

### Biological material

The strain *Nannochloropsis oceanica* NCT02 was kindly provided by Necton S.A. (Algarve, Potugal). Necton S.A. is willing to provide the strain on request.

## Results and Discussion

*Nannochloropsis oceanica* production can aim at different final products. The whole biomass of microalgae is rich in pigments and fatty acids, but also has high valuable proteins and carbohydrates. Depending on the end-product desired, the production of the biomass can be tuned to reach higher yields. For that reason, the experiments of this work were designed to increase the concentration range of three different products, biomass (as cell concentration), chlorophyll and fatty acids. Having a wider range of scenarios increases the range of the outputs, which results in an increased strength of the prediction models. The information about the experimental conditions tested and the respective cell concentration, chlorophyll and fatty acids measurements are available in Supplementary Information.

**Figure 1:**
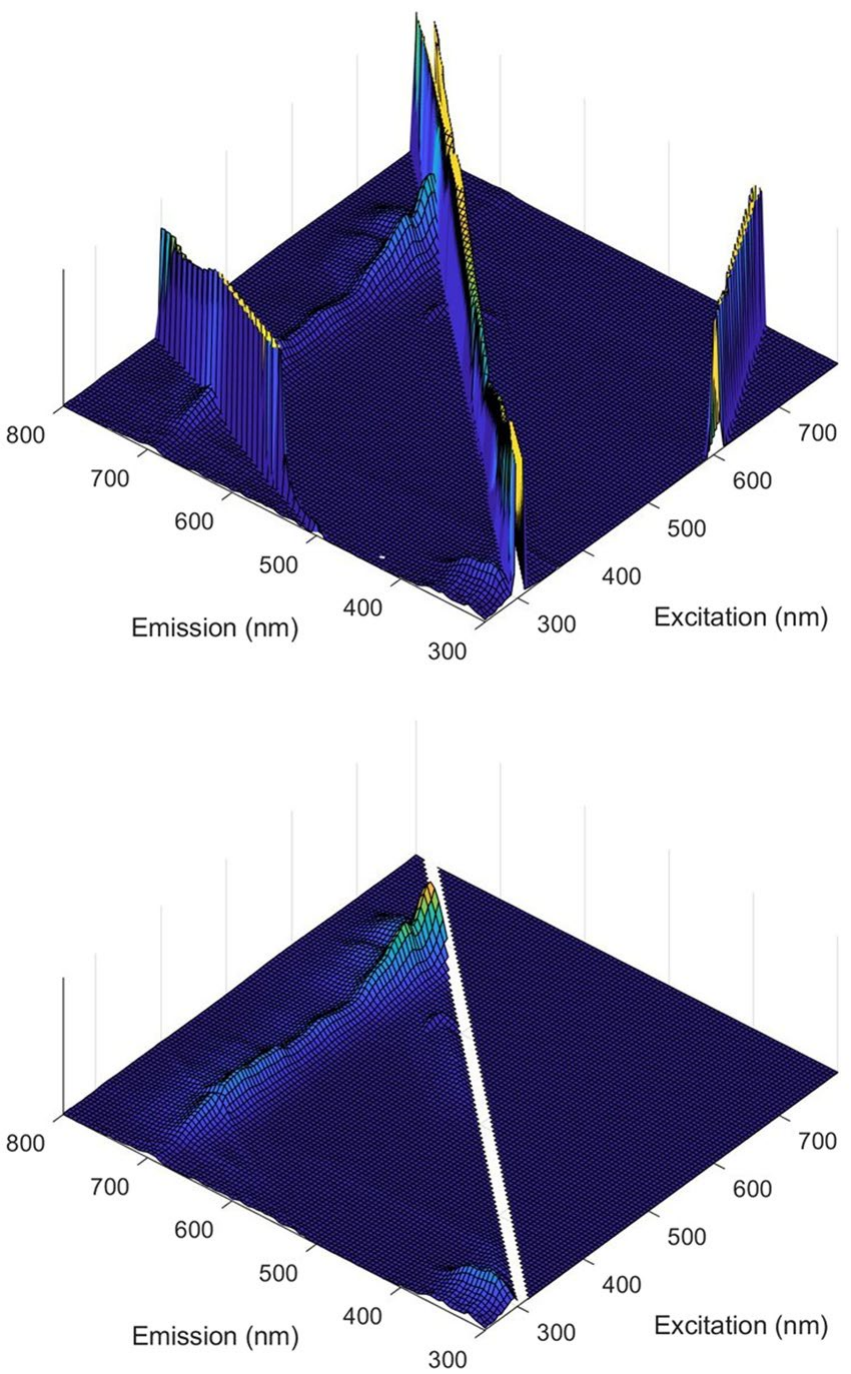
Fluorescence spectra of a *Nannochloropsis oceanica* sample: original spectra (**a**) and final spectra used as inputs in the PLS models (**b**). Rayleigh scatter of first order was removed and replaced by empty values; the second order was replaced with an interpolation of surrounding data points. Fluorescence signal corresponding to emission wavelengths (y-axis) shorter than the excitation wavelengths (x-axis) was replaced by zeros. Inner filter effects were also corrected whenever present.

Microalgae cultivation is characterised by low biomass concentrations, mainly to avoid dark zones in the bioreactors that can lead to lower photosynthetic efficiencies^29^. For this reason, microalgae samples have high water content, that results in the presence of high intensity Rayleigh scatter (Fig. 1a). The presence of water scatter would impact the estimation of the regression coefficients of the final models (presented in section 3.4)^30^, and since its signal is not proportional to the water content in the sample, it was removed before the PLS modelling, as described in Section 2.3. An example of the pre-processed spectra is shown in Fig. 1b.

### Cell concentration

**Figure 2:**
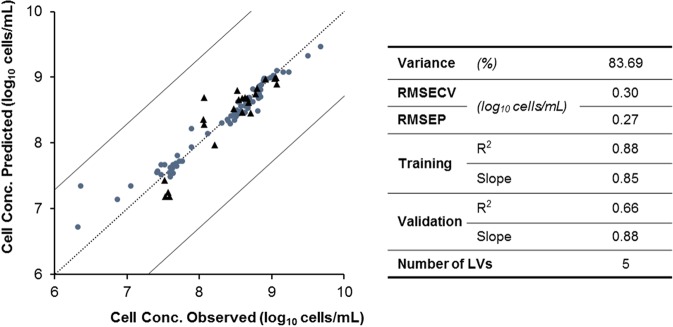
Cell concentration prediction model (one of the four partitions of training/validation data sets). Training (●) (n = 69) and validation (▲) (n = 23) data are represented in log_10_ cells/mL. Model performance parameters: variance captured (Variance); root mean square error of cross-validation (RMSECV); root mean square error of prediction (RMSEP); coefficients of determination (R^2^) and slopes of linear regression between observed and predicted data obtained respectively for the training and validation data sets; number of LVs used by the model.

The model obtained to monitor cell concentration of *N. oceanica* can explain 84% of variance captured by the fluorescence spectroscopy (Fig. 2) with five LVs. A low root mean square error of *N. oceanica* concentration prediction (RMSEP) was observed (0.27 log_10_ cells/mL), which represents the average distance between the observed values and the ones predicted by the model. The relative error (in percentage), calculated as a quotient between the prediction error (RMSEP) and the observed cell concentration average value, is 3.18%. The root mean square error of cross-validation (RMSECV) of 0.30 log_10_ cells/mL indicates absence of model overfit. The reported R^2^ for training refers to the model built on the whole training set, whereas the validation data was never used for any step of model building and therefore tends to have a lower R^2^. Furthermore, the observed lack-of-fit is mainly due to the poor predictions for samples with lowest cellular concentrations, see Fig. 2.

In the current industrial scenario, most microalgal products are sold as whole biomass powder, making total biomass a key parameter to control process efficiency. During cultivation at industrial scale, too high or too low biomass concentration can have an influence in several biological parameters. Low concentrations can result in an inefficient light absorption or photo inhibition, while high concentrations result in dark regions in the bioreactor triggering endogenous respiration^29^.

Biomass concentration can be monitored under different parameters, such as optical density (OD), dry weight (g/L) and cell concentration (cells/L). In the present work, biomass concentration was measured as cell concentration. The main reason was because the experiments were designed to give a wide range of cell concentration, but some experiments induce also other biological changes, such as different coloration or accumulation of fatty acids. For example, as mentioned by Janssen *et al*.^31^, accumulation of fatty acids in lipid bodies, due to nitrogen depletion medium, leads to an increase of the dry weight while the cell concentration reaches a plateau. This phenomenon was also observed with the experiments of this work (data not shown). Experiments using day/night cycle lead the biomass to follow circadian rhythms, which means that the cell size increases during the day while the cell concentration increases during the night (due to cell division)^32,33^.

A previous study reported the use of fluorescence spectroscopy to monitor cell concentration of a different microalgae, *Dunaliella salina*^34^. A slightly different modelling approach was used then, since PCA (principal component analysis) was performed on the EEMs, without the need to remove the scatter, prior to PLS modelling. Nevertheless, a similar explained variance was observed (between 85.7 and 86.3%), with similar values of R^2^ for training and validation sets (between 0.82 and 0.86). These results, together with the results of this work, demonstrate the potential of using fluorescence spectroscopy as a monitoring tool for cell concentration with different microalgae biomass.

### Chlorophyll

Chlorophyll is the most abundant light harvesting pigment in nature, enabling the photosynthesis, and is a molecule well-studied for its potential in several fields. In the feed and food supplements industry, chlorophyll is relevant for its anti-oxidant properties. Because of its bright green colour, it is also an appealing dye for the food and paint industries^13,14^.

Chlorophyll content in microalgae is tightly correlated with the light intensity and circadian rhythms. It was reported that chlorophyll content increases during the light period, and starts to decrease with the beginning of a dark period^32,33,35^. That phenomenon is explained by the fact that the cell division mechanism is favourable in the dark period, and since *Nannochloropsis* genus divide by binary fission, the chlorophyll content of the “adult” cell is divided by its new cells^33^.

The experimental conditions induced a considerable variability in the chlorophyll content. As mentioned previously, day/night cycles induce the chlorophyll to oscillate during the experiments. Moreover, microalgae are known for their ability to adapt their photosynthetic apparatus to different light conditions, a process called photoacclimation. High light intensities reduce chlorophyll content to protect the cell, while low intensities induced the photosynthetic apparatus to synthesise chlorophyll, to provide the cell with more light harvesting capacity^31,36^. Nitrogen starvation, however decreases chlorophyll content and increases carotenoid concentrations to equip the cell with stress defence mechanisms^31,33^.

**Figure 3:**
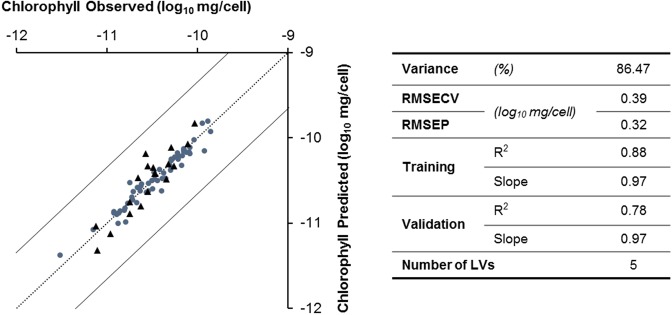
Chlorophyll content prediction model (one of the four partitions of training/validation data sets). Training (●) (n = 57) and validation (▲) (n = 19) data are represented in log_10_ mg/cell. Model performance parameters: variance captured (Variance); root mean square error of cross-validation (RMSECV); root mean square error of prediction (RMSEP); coefficients of determination (R^2^) and slopes of linear regression between observed and predicted data obtained respectively for the training and validation data sets; number of LVs used by the model.

The chlorophyll content studied in these experiments enabled the development of an accurate model (Fig. 3), with a relative error of 1.31% using five LVs. The validation and training sets show high R^2^ and low errors, both RMSECV and RMSEP.

The possibility of monitoring chlorophyll content online provides information about the physiological state of the microalgae at real time. With this knowledge it is possible to take decisions during the cultivation process, without the need to perform the time-consuming lab analysis that are usually required.

### Lipids

Several microalgae are being studied for their potential to produce high content and/or high-quality lipids. Having in mind the different opportunities for an enriched-lipid biomass, the *N. oceanica* lipid profile was assessed through a set of different experiments. Nitrogen depletion is a well-documented strategy to increase lipid content in the TAG fraction^10,37^, while low light conditions were documented to lead to an increase in the cellular membranes^37^. Also, low temperatures were described to increase the content of unsaturated fatty acids^10,38,39^, whereas high temperature favour the saturated^40^.

**Figure 4:**
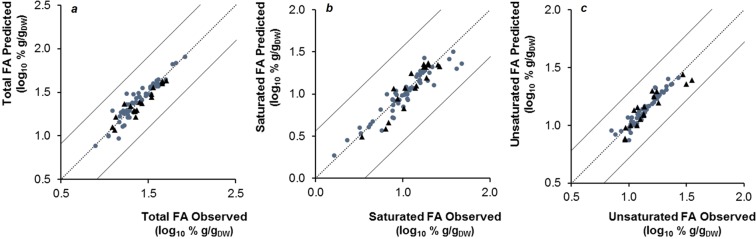
Fatty acids (FA) prediction models for total (**a**), saturated (**b**) and unsaturated (**c**) FA (one of the four partitions of training/validation data sets). Training (●) (n = 54) and validation (▲) (n = 18) data are represented in log_10_% g/g _DW_.

Models were obtained to monitor fatty acids as total content (Fig. 4a), as well as saturated (Fig. 4b) and unsaturated (Fig. 4c) content only (Table 2). Values of explained variance ranged between 87 and 92%. When compared with the previous models of this work, a higher number of LVs is needed (between 9 and 10). The relative error of prediction for total and unsaturated fatty acids was 5.89% and 5.62%, respectively, lower than the value found for saturated fatty acids (9.54%). All R^2^ of validation and training set were above 0.74 and slopes close to 1.

**Table 2:** Prediction model parameters for total, saturated and unsaturated fatty acids.

		Total	Saturated	Unsaturated
Variance	*(%)*	92.30	91.24	86.77
RMSECV	*(log*_*10%*_ *g/g*_*DW*_*)*	0.21	0.29	0.17
RMSEP		0.19	0.23	0.15
Training	R^2^	0.87	0.90	0.85
	Slope	0.92	0.93	0.95
Validation	R^2^	0.78	0.81	0.74
	Slope	0.84	0.95	0.99
Number of LVs		10	10	9

Model performance parameters: variance captured (Variance); root mean square error of cross-validation (RMSECV); root mean square error of prediction (RMSEP); coefficients of determination (R2) and slopes of linear regression between observed and predicted data obtained respectively for the training (n = 54) and validation (n = 18) data sets; number of LVs (latent variables) used by the model.

When cultivated under optimal growing conditions, most of the microalgae lipids are present on the cellular membrane^11^. However, under stress growing conditions some microalgae, like *Nannochloropsis*, can accumulate up to 45% of their dry weight in triacylglycerol (TAG)^10,37^. According to the fraction of the cell where the lipid is accumulated and its profile, the final destination of the lipid-enriched biomass can vary from feed or food supplements to biodiesel production^11,41,42^.

Fatty acids can be classified into saturated or unsaturated, the latter into mono- or polyunsaturated according to the number of double chemical bonds. Biomass produced with the aim of supplying feed or food supplements industries is desired to be rich in unsaturated fatty acids, preferably omega-3 fatty acids such as EPA (eicosapentaenoic acid) or DHA (docosahexaenoic acid)^11,37^. Biomass produced for biofuel applications needs to fulfil quality parameters such as ignition and combustion performance, and those are directly correlated with saturated and unsaturated content^10,43^.

To our knowledge, fluorescence spectroscopy was not previously reported as an online monitoring tool for lipid content. It is known that fluorescence spectroscopy is highly sensitive to detect the presence of natural fluorophores and that lipid molecules are not natural fluorophores. In the presence of a complex matrix, like microalgae cultivation broth, fluorescence spectroscopy is able not only to identify the natural fluorophores (intra or extracellular) but also the relations between these compounds and the non-fluorophores^9^. For that reason, fluorescence spectroscopy spectra cannot be directly used for quantification, but is a powerful tool revealing the correlations between compounds that emit natural fluorescence, the ones that capture it, and the ones that somehow mask the fluorescence signal.

### Regression coefficients of the final models for cell concentration, chlorophyll and fatty acids

The experiments performed enabled to acquire a wide range of scenarios of a *N. oceanica* cultivation for different end products. After calibrating the models for the parameters cell concentration, chlorophyll and fatty acids, it is possible to confirm that fluorescence spectroscopy has a great potential for online monitoring of all three parameters simultaneously.

**Figure 5:**
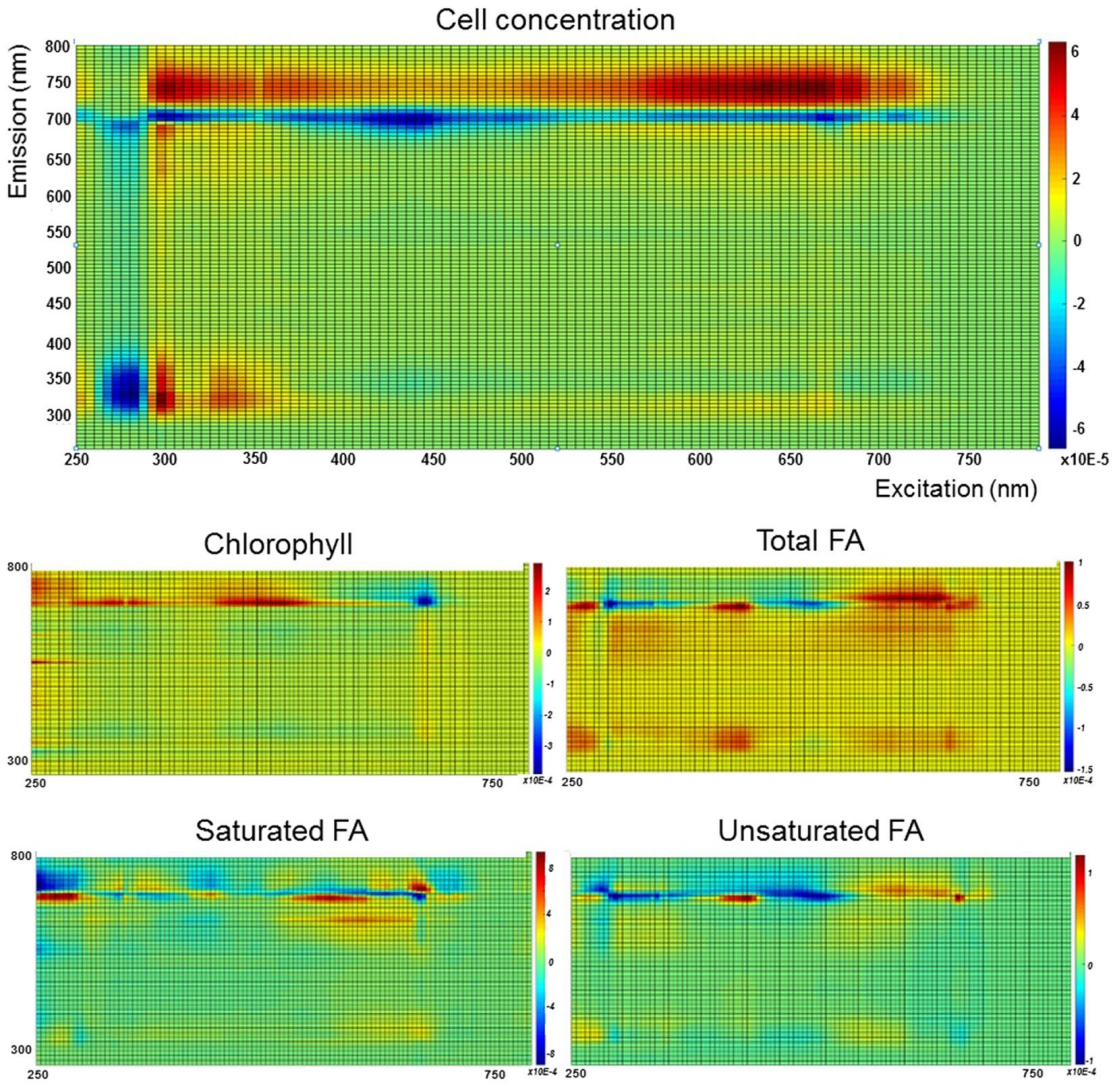
Regression coefficients of the prediction models for cell concentration, chlorophyll, and fatty acids (FA) as total, saturated and unsaturated. The training set used 100% of the data set. Excitation wavelengths are represented in the x-axis, emission wavelengths in the y-axis, and intensity is represented in the colour bar on the right side.

Aiming at the application of fluorescence spectroscopy, a final model was created for each parameter studied, using 100% of the data as training set. Figure 5 shows the regression coefficients obtained for each output, where the excitation and emission wavelengths (in nm) are in the x-axis and y-axis, respectively, and each square is an excitation/emission pair. The weight of each regression coefficient is represented in colour-scale.

For the cell concentration model, two main regions can be distinguished as having relevant regression coefficient weight (positive or negative): a band at emission wavelengths higher than 600 nm (whole excitation wavelength range), and a region for excitation and emission wavelengths lower than 400 nm. This reveals a similarity with the overall fluorescence signal of a sample, where these two regions have high fluorescence intensities. As described previously, these two regions of the spectra correspond to the pigments fluorescence band and the protein-like region (aromatic aminoacids), respectively^34,44–46^. Several differences can be noticed between the regression coefficients map of the cell concentration model and the remaining outputs. The protein-like region does not have the same weight as for cell concentration, and different weights are attributed to the pigments band. As expected, a high correlation is shown between the pigments band and the regression coefficient map for chlorophyll content prediction. Also, specific areas of this pigment band are used for fatty acids content prediction (as total, saturated or unsaturated). These results confirm the relationship previously described between chlorophyll and fatty acids content^33^, and that although fatty acids do not emit fluorescence, they interfere with the signal of natural fluorophores like chlorophyll. The development of a simpler spectrofluorometric technique, able to acquire signal in those two regions of the spectra, instead of the entire range, would possibly simplify the analysis by decreasing the acquisition time of each data point.

Using fluorescence spectroscopy to simultaneously monitor several biological parameters has been described as one of the powerful characteristics of this technique^9^. By coupling the fluorescence EEMs and the regression coefficients developed by chemometric models, more knowledge about the cultivation process can be acquired, enabling important decisions at real time, like the optimum harvesting time.

## Conclusions

The present work demonstrates the feasibility of using fluorescence spectroscopy coupled to chemometrics to assess multiple parameters during *N. oceanica* cultivation. It was shown that this technique can be applied to this microalga to assess not only biomass (as cell concentration) and pigments (chlorophyll), as in previous studies, but also different fractions of fatty acids. This outcome has a major impact on the monitoring of microalgae production, especially when aiming lipids production.

Different environmental conditions were tested to increase the response range of the parameters under study, which allowed the development of accurate models for all the parameters with low errors (REMSECV and RMSEP between 0.12 and 0.40) and high R^2^ (between 0.65 and 0.93). Furthermore, the regression coefficient maps highlight the importance of the pigment and the protein regions for the development of these models.

In overall, the fluorescence excitation-emission matrices contain a huge amount of diverse information from the process that can be translated into quantitative information using adequate mathematical tools, such as developed in this study.

## Supplementary information

Supplementary Information.

## Data Availability

The datasets generated and analysed during the current study will be available in the MAGNIFICENT Zenodo repository, after publication.
