# Supplementary material for: Fluorescence spectroscopy and chemometrics for simultaneous monitoring of cell concentration, chlorophyll and fatty acids in Nannochloropsis oceanica
Source: Sci Rep. 2020 May 6;10:7688. doi: 10.1038/s41598-020-64628-7 (PMC7203222; doi:10.1038/s41598-020-64628-7)
Supplement: Supplementary file 1 — Supplementary Information. [file 41598_2020_64628_MOESM1_ESM.pdf]

Supplementary Information

Fluorescence spectroscopy and chemometrics for simultaneous monitoring of cell concentration, chlorophyll and fatty acids in *Nannochloropsis oceanica*

Marta Sá[1,2]

Carlo G. Bertinetto[3]

Narcís Ferrer-Ledo[2]

Jeroen J. Jansen[3]

Rene Wijffels[2]

João G. Crespo[1]

Maria Barbosa[2]

Claudia F. Galinha[1]*

[1] LAQV-REQUIMTE, Chemistry Department, FCT, Universidade Nova de Lisboa, Caparica, Portugal

[2] Bioprocess Engineering, AlgaePARC, Wageningen University and Research, Wageningen, Netherlands

[3] Radboud University, Department of Analytical Chemistry and Chemometrics, Institute for Molecules and Materials, Nijmegen, Netherlands

Correspondence to: Claudia F. Galinha, LAQV-REQUIMTE, Chemistry Dept, FCT, Universidade Nova de Lisboa, Quinta da Torre, 2829-516 Caparica, Portugal

E-mail: cf.galinha@fct.unl.pt

Table S1. *Cell concentration (in cells/mL and log_{10}), chlorophyll (in mg/cell and log_{10}) and fatty acids content (in mg/cell and log_{10}), measured in Nannochloropsis oceanica cultivation experiments, used for the calibration and validation of the chemometric models. Four different temperatures were studied (15, 20, 25 and 30 °C), 24h of light was provided (d (24)), and nitrogen depletion strategy (N-starv √) was performed. Detailed information about the cultivation experiments can be found in Sá et al. (2020).*

Batch information				Cell Concentration		Chorophyll		Fatty acids					
								Total		Saturated		Unsaturated	
Temp (°C)	N-starv	Light (hours)	Day	(cells/mL)	*(log_{10})*	(mg/cell)	*(log_{10})*	(% g/g_{DW})	*(log_{10})*	(% g/g_{DW})	*(log_{10})*	(% g/g_{DW})	*(log_{10})*
			**1**	1.85E+07	7.27								
			**2**	1.77E+07	7.25	9.89E-11	-10.00	25.74	3.25	11.69	2.26	14.04	2.64
			**3**	2.00E+07	7.30			27.58	3.32	13.08	2.42	14.50	2.67
			**4**	2.94E+07	7.47	6.25E-11	-10.20	27.67	3.32	13.21	2.36	14.46	2.67
			**5**	3.19E+07	7.50								
			**6**	6.93E+07	7.84	4.48E-11	-10.35						
			**7**	1.09E+08	8.04								
**15**	√	**d (24)**	**8**	1.60E+08	8.20	3.40E-11	-10.47	28.59	3.35	12.52	2.35	16.07	2.78
			**9**	2.98E+08	8.47			29.98	3.40	13.34	2.41	16.64	2.81
			**10b**	5.86E+08	8.77	1.29E-11	-10.89						
			**10**	2.22E+08	8.35			32.95	3.50	14.41	2.51	18.54	2.92
			**11**	2.98E+08	8.47			36.41	3.59	16.17	2.66	20.25	3.01
			**12**	3.29E+08	8.52	7.53E-12	-11.12	42.16	3.74	19.12	2.84	23.04	3.14
			**14**	3.64E+08	8.56	4.81E-12	-11.32	45.35	3.81	20.69	2.95	24.67	3.21
			**15**	3.92E+08	8.59			50.46	3.92	22.76	3.04	27.70	3.32
			**1**	1.52E+07	7.18	8.42E-11	-10.07						
			**2**	1.54E+07	7.19	5.65E-11	-10.25	21.96	3.09	10.22	2.11	11.73	2.46
			**3**	2.69E+07	7.43	5.93E-11	-10.23	26.34	3.27	12.55	2.37	13.78	2.62
			**4**	5.04E+07	7.70	4.90E-11	-10.31	29.28	3.38	14.03	2.48	15.25	2.72
			**5**	9.44E+07	7.98	4.40E-11	-10.36	24.19	3.19	11.30	2.26	12.89	2.56
**20**	√	**d (24)**	**6**	2.04E+08	8.31	1.57E-11	-10.80						
			**7b**	2.90E+08	8.46	3.60E-11	-10.44						
			**7**	2.31E+08	8.36	3.44E-11	-10.46	24.87	3.21	11.84	2.31	13.02	2.57
			**8**	4.63E+08	8.67	1.76E-11	-10.75	30.69	3.42	15.01	2.59	15.68	2.75
			**9**	5.32E+08	8.73	1.36E-11	-10.87	31.36	3.45	15.61	2.66	15.75	2.76
			**10**	6.40E+08	8.81	8.78E-12	-11.06	42.09	3.74	21.08	2.97	21.01	3.05
			**11**	5.56E+08	8.75	8.52E-12	-11.07	42.32	3.75	21.02	2.98	21.30	3.06
			**1**	1.69E+07	7.23	1.20E-10	-9.92						
			**2**	5.04E+07	7.70	5.44E-11	-10.26	23.35	3.15	11.85	2.24	11.50	2.44
			**3**	1.49E+08	8.17	6.21E-11	-10.21	16.56	2.81	6.86	1.46	9.69	2.27
**25**	√	**d (24)**	**4**	5.56E+08	8.75			19.55	2.97	9.36	1.89	10.19	2.32
			**5**	5.83E+08	8.77	1.61E-11	-10.79	32.99	3.50	17.07	2.70	15.91	2.77
			**6**	5.57E+08	8.75	1.42E-11	-10.85	40.10	3.69	21.37	2.97	18.73	2.93
			**7**	6.10E+08	8.79	1.06E-11	-10.97	41.37	3.72	22.44	3.04	18.93	2.94
			**8**	6.30E+08	8.80	9.23E-12	-11.03	43.21	3.77	23.26	3.07	19.96	2.99
			**1**	9.75E+06	6.99	1.33E-10	-9.88						
			**2**	2.39E+07	7.38	5.11E-11	-10.29	21.69	3.08	11.59	2.27	10.10	2.31
			**3**	7.50E+07	7.88	4.62E-11	-10.34	19.73	2.98	9.70	2.02	10.02	2.30
			**4**	1.73E+08	8.24			11.03	2.40	3.77	0.44	7.26	1.98
			**5b**	5.68E+08	8.75								
**25**	√	**d (24)**	**5**	3.43E+08	8.54	4.86E-11	-10.31	19.75	2.98	8.82	1.79	10.94	2.39
			**6**	4.79E+08	8.68	3.29E-11	-10.48	33.55	3.51	17.25	2.72	16.31	2.79
			**7**	4.83E+08	8.68	2.31E-11	-10.64	41.78	3.73	21.96	3.01	19.82	2.99
			**8**	5.42E+08	8.73	1.93E-11	-10.71	41.84	3.73	22.13	3.02	19.70	2.98
			**9**	5.24E+08	8.72	1.88E-11	-10.73	40.67	3.71	21.49	3.00	19.18	2.95
			**10**	6.78E+08	8.83	1.45E-11	-10.84	40.95	3.71	21.78	3.01	19.17	2.95
			**11**	5.86E+08	8.77	1.33E-11	-10.87	41.90	3.74	22.21	3.03	19.69	2.98
			**1**	2.00E+07	7.30	5.87E-11	-10.23						
			**2**	4.66E+07	7.67	4.76E-11	-10.32	22.43	3.11	11.16	2.20	11.27	2.42
			**3**	1.36E+08	8.13	5.03E-11	-10.30	23.19	3.14	11.55	2.24	11.63	2.45
			**4b**	5.01E+08	8.70	4.48E-11	-10.35						
**30**	√	**d (24)**	**4**	2.85E+08	8.46	3.79E-11	-10.42	20.92	3.04	10.13	2.03	10.79	2.38
			**5**	3.19E+08	8.50	2.91E-11	-10.54	35.77	3.58	18.06	2.79	17.71	2.87
			**6**	4.56E+08	8.66	1.45E-11	-10.84	39.15	3.67	19.71	2.90	19.45	2.97
			**7**	4.17E+08	8.62	1.15E-11	-10.94	46.09	3.83	23.22	3.08	22.87	3.13
			**8**	4.67E+08	8.67	8.32E-12	-11.08	45.33	3.81	21.93	3.02	23.40	3.15

Nannochloropsis oceanica stress induction, by nitrogen depletion, was performed in different days for the different experiments. In those days, samples were taken before (day-'b') and after changing the media from nitrogen replete to nitrogen deplete.

Table S2. *Cell concentration (in cells/mL and log₁₀), chlorophyll (in mg/cell and log₁₀) and fatty acids content (in mg/cell and log₁₀), measured in Nannochloropsis oceanica cultivation experiments, used for the calibration and validation of the chemometric models. The experiments were performed under day/night cycle, 16h of light and 8h of dark (d/n (16/8)). Two different studies were performed: keep the temperature at 25 °C and study nitrogen depletion (N-starv √); decrease the temperature from 25 to 15 °C (25→15 °C)keeping the medium nitrogen depleted (N-starv x). Detailed information about the cultivation experiments can be found in Sá et al. (2020).*

Batch information				Cell Concentration		Chorophyll		Fatty acids					
Temp (°C)	N-starv	Light (hours)	Day	(cells/mL)	*(log 10)*	(mg/cell)	*(log 10)*	Total (% g/g$_{DW}$)	Total *(log 10)*	Saturated (% g/g$_{DW}$)	Saturated *(log 10)*	Unsaturated (% g/g$_{DW}$)	Unsaturated *(log 10)*
			1	1.19E+07	7.08	9.12E-11	-10.04	23.74	3.17	10.03	2.00	13.71	2.62
			2	1.53E+07	7.19	4.76E-11	-10.32						
			4	9.62E+07	7.98	7.71E-11	-10.11						
			5	3.50E+08	8.54			18.76	2.93	7.25	1.59	11.51	2.44
			6b	4.93E+08	8.69								
			6	4.15E+08	8.62	5.80E-11	-10.24	19.03	2.95	6.73	1.08	12.30	2.51
25	√	d/n (16/8)	7	7.83E+08	8.89	2.64E-11	-10.58	23.66	3.16	10.08	1.88	13.58	2.61
			8	7.82E+08	8.89	2.07E-11	-10.68	27.46	3.31	13.09	2.30	14.36	2.66
			10	9.68E+08	8.99	1.28E-11	-10.89	34.72	3.55	17.76	2.70	16.96	2.83
			12	9.48E+08	8.98	1.35E-11	-10.87	40.41	3.70	21.30	2.92	19.11	2.95
			14	9.55E+08	8.98	9.11E-12	-11.04	42.41	3.75	22.52	2.99	19.89	2.99
			16	9.95E+08	9.00	7.32E-12	-11.14	45.70	3.82	24.17	3.09	21.54	3.07
			21	1.11E+09	9.05	1.26E-11	-10.90	48.83	3.89	26.11	3.11	22.72	3.12
			1	1.52E+07	7.18	1.47E-10	-9.83						
			3	6.75E+07	7.83	1.02E-10	-9.99	16.55	2.81	7.17	1.56	9.38	2.24
			4	1.91E+08	8.28	8.70E-11	-10.06						
			5	4.92E+08	8.69			16.47	2.80	6.64	1.45	9.83	2.29
			6	5.18E+08	8.71	7.36E-11	-10.13	13.29	2.59	4.77	0.79	8.52	2.14
25	x	d/n (16/8)	7	7.14E+08	8.85			11.97	2.48	3.87	0.15	8.10	2.09
			8	9.75E+08	8.99	3.63E-11	-10.44	11.64	2.45	3.56	-0.31	8.08	2.09
			9	1.07E+09	9.03	4.69E-11	-10.33	11.16	2.41	3.40	-0.81	7.76	2.05
			11	1.11E+09	9.05	6.51E-11	-10.19	11.77	2.47	3.67	-0.72	8.10	2.09
			13	1.51E+09	9.18	3.77E-11	-10.42	11.62	2.45	3.88	-0.30	7.74	2.05
			15	1.51E+09	9.18	3.36E-11	-10.47	11.49	2.44	3.92	-0.16	7.57	2.02
			1	1.21E+07	7.08	1.13E-10	-9.95						
			3	3.44E+07	7.54	9.52E-11	-10.02	22.77	3.13	10.33	1.93	12.44	2.52
			4	8.02E+07	7.90	6.81E-11	-10.17	26.70	3.28	12.88	2.32	13.82	2.63
			5	2.30E+08	8.36	3.86E-11	-10.41	20.77	3.03	8.79	1.84	11.98	2.48
			6	4.46E+08	8.65	4.32E-11	-10.36	21.31	3.06	9.24	1.96	12.07	2.49
25 → 15	x	d/n (16/8)	7	4.75E+08	8.68	3.88E-11	-10.41	22.08	3.09	9.12	1.94	12.95	2.56
			8	4.84E+08	8.69	4.70E-11	-10.33	19.39	2.96	7.22	1.63	12.17	2.50
			9	5.21E+08	8.72	5.99E-11	-10.22	15.76	2.76	4.59	0.97	11.17	2.41
			11	6.31E+08	8.80	2.36E-11	-10.63	13.64	2.61	3.65	0.54	9.98	2.30
			13	1.67E+09	9.22	2.52E-11	-10.60	13.29	2.59	3.37	0.27	9.93	2.30
			15	1.81E+09	9.26	3.04E-11	-10.52	12.60	2.53	3.09	0.07	9.50	2.25
			17	2.11E+09	9.33	3.45E-11	-10.46	13.12	2.57	3.21	0.12	9.92	2.29

Nannochloropsis oceanica stress induction, by nitrogen depletion or temperature decrease, was performed in different days for the different experiments. When samples were taken before and after stress induction, 'b' marks the samples taken before the stress induction.

References

Sá, M., Ferrer-Ledo, N., Wijffels, R., Crespo, J. G., Barbosa, M., & Galinha, C. F. (2020). Monitoring of eicosapentaenoic acid (EPA) production in the microalgae Nannochloropsis oceanica. *Algal Research*, *45*, 101766. https://doi.org/10.1016/J.ALGAL.2019.101766
